# Dye-Mediated Photo-Oxidation Biomaterial Fixation: Analysis of Bioinductivity and Mechanical Properties of Bovine Pericardium for Use in Cardiac Surgery

**DOI:** 10.3390/ijms221910768

**Published:** 2021-10-05

**Authors:** Simranjit S. Pattar, Vishnu Vasanthan, Guoqi Teng, Karl T. Wagner, Kristina Jeon, Sean Kang, Ali Fatehi Hassanabad, Paul W. M. Fedak

**Affiliations:** Department of Cardiac Sciences, Libin Cardiovascular Institute, Cumming School of Medicine, University of Calgary, Calgary, AB T2N 1N4, Canada; sspattar@ucalgary.ca (S.S.P.); vishnu.vasanthan@ucalgary.ca (V.V.); teng@ucalgary.ca (G.T.); ktwagner@ualberta.ca (K.T.W.); kjeon@ualberta.ca (K.J.); sekang@ucalgary.ca (S.K.); ali.fatehihassanabad@albertahealthservices.ca (A.F.H.)

**Keywords:** cardiac surgery, biomaterial, tissue fixation, cardiac fibroblast, myocardial infarction, cardiac reconstruction, post-infarct cardiac repair, vasculogenesis, dye-mediated photo-oxidation

## Abstract

Extracellular matrix bioscaffolds can influence the cardiac microenvironment and modulate endogenous cellular mechanisms. These materials can optimize cardiac surgery for repair and reconstruction. We investigated the biocompatibility and bioinductivity of bovine pericardium fixed via dye-mediated photo-oxidation on human cardiac fibroblast activity. We compared a dye-mediated photo-oxidation fixed bioscaffold to glutaraldehyde-fixed and non-fixed bioscaffolds reported in contemporary literature in cardiac surgery. Human cardiac fibroblasts from consenting patients were seeded on to bioscaffold materials to assess the biocompatibility and bioinductivity. Human cardiac fibroblast gene expression, secretome, morphology and viability were studied. Dye-mediated photo-oxidation fixed acellular bovine pericardium preserves human cardiac fibroblast phenotype and viability; and potentiates a pro-vasculogenic paracrine response. Material tensile properties were compared with biomechanical testing. Dye-mediated photo-oxidation fixed acellular bovine pericardium had higher compliance compared to glutaraldehyde-fixed bioscaffold in response to tensile force. The biocompatibility, bioinductivity, and biomechanical properties of dye-mediated photo-oxidation fixed bovine pericardium demonstrate its feasibility as a bioscaffold for use in cardiac surgery. As a fixed yet bioinductive solution, this bioscaffold demonstrates enhanced compliance and retains bioinductive properties that may leverage endogenous reparative pathways. Dye-mediated photo-oxidation fixed bioscaffold warrants further investigation as a viable tool for cardiac repair and reconstruction.

## 1. Introduction

Cardiac surgery is a heavily reconstructive specialty that relies on glutaraldehyde (GA) fixed bovine pericardium for robust and reproducible repair, in a variety of cases. GA-fixed bovine pericardium is widely used because it is able to provide robust structural support and does not rapidly break down after implantation; but it is prone to calcification and inflammation, in addition to glutaraldehyde cytotoxicity [1,2,3,4,5,6]. There is a need for surgical biomaterials prepared with novel fixation strategies that maintain structural support and resistance to breakdown, while also providing a hospitable biocompatible environment to promote repair at a cellular level.

The use of decellularized extracellular matrix bioscaffolds in cardiac surgery is promising, given the broad application of these bioscaffolds in cardiac structural repair and myocardial regeneration. It is increasingly apparent that the properties of each bioscaffold, including tissue source, fixation method, immunogenic properties, growth factor content, and biomechanical properties, determine its suitability for specific clinical scenarios in cardiac repairs at the time of cardiac surgery [7,8]. Our group’s previous work explored non-fixed acellular porcine small intestinal submucosal extracellular matrix (SIS-ECM) and its ability to influence the local cardiac microenvironment. This material modulated cellular activity and enhanced endogenous mechanisms of repair by attenuating cardiac fibrosis, promoting angiogenesis and ultimately improving post-infarct cardiac function in small and large animal models [9,10,11]. Furthermore, glutaraldehyde fixation of SIS-ECM caused loss of bioinductivity [10]. Despite the benefits of non-fixed acellular bioscaffolds, challenges regarding resistance against breakdown, immunogenicity, and inflammation, seen particularly in pediatric applications, remain hurdles to clinical translation [12,13,14].

Though glutaraldehyde-fixed bovine pericardium is commonly used in cardiac surgery for structural repair, there is currently no gold standard. The ideal biomaterial for cardiac surgery would provide predictability and robustness of a traditionally fixed bioscaffold, but also modulate the local cardiac environment similarly to non-fixed bioactive ECM. Herein, we investigate a commercially available acellular bovine pericardium bioscaffold fixed via dye-mediated photo-oxidation (DMPO-fixed ECM bioscaffold; PhotoFix; CryoLife Inc., Kennesaw, GA, USA). We assess material bioinductivity and its ability to modulate the activity and phenotype of cardiac fibroblasts, which are key modulators of cardiac remodeling [15,16]. We compared DMPO-fixed ECM to the widely used glutaraldehyde (GA)-fixed ECM bioscaffold (Peri-Guard; Synovis, MN, USA) in terms of fibroblast transcriptome, fibroblast angiogenic paracrine activity, fibroblast morphology and viability; and tensile structural strength was also compared. Additionally, given the hypothesized bioinductivity of this material, we compared the paracrine activity of human cardiac fibroblasts seeded on DMPO-fixed bovine pericardium with those seeded on known bioactive SIS-ECM (Cor^TM^ PATCH; CorMatrix Cardiovascular Inc., Roswell, GA, USA). Our findings show that DMPO-fixed ECM provides a hospitable environment for cardiac fibroblasts compared to GA-fixed ECM. DMPO-fixed ECM modulates fibroblast paracrine activity to promote vasculogenic pathways similarly to SIS-ECM, suggesting its potential for cardiac tissue repair.

## 2. Results

### 2.1. DMPO-Fixed ECM Bioscaffold Modulates Transcription of Vasculogenic, Fibrogenic, And Extracellular Matrix Remodeling Pathways in Human Cardiac Fibroblasts

Though glutaraldehyde fixed bovine pericardium is widely used, DMPO-fixed bovine pericardium may be more biocompatible and less cytotoxic; and it may also promote reparative pathways. RNA sequencing assessed if DMPO-fixed ECM could maintain healthy human cardiac fibroblast function when seeded on the bioscaffold. Cardiac fibroblasts were seeded on DMPO-fixed or glutaraldehyde (GA)-fixed ECM for 24 h, followed by RNA sequencing analysis to compare differential gene expression regarding vasculogenic, fibrogenic, and extracellular matrix remodeling pathways.

**Figure 1:**
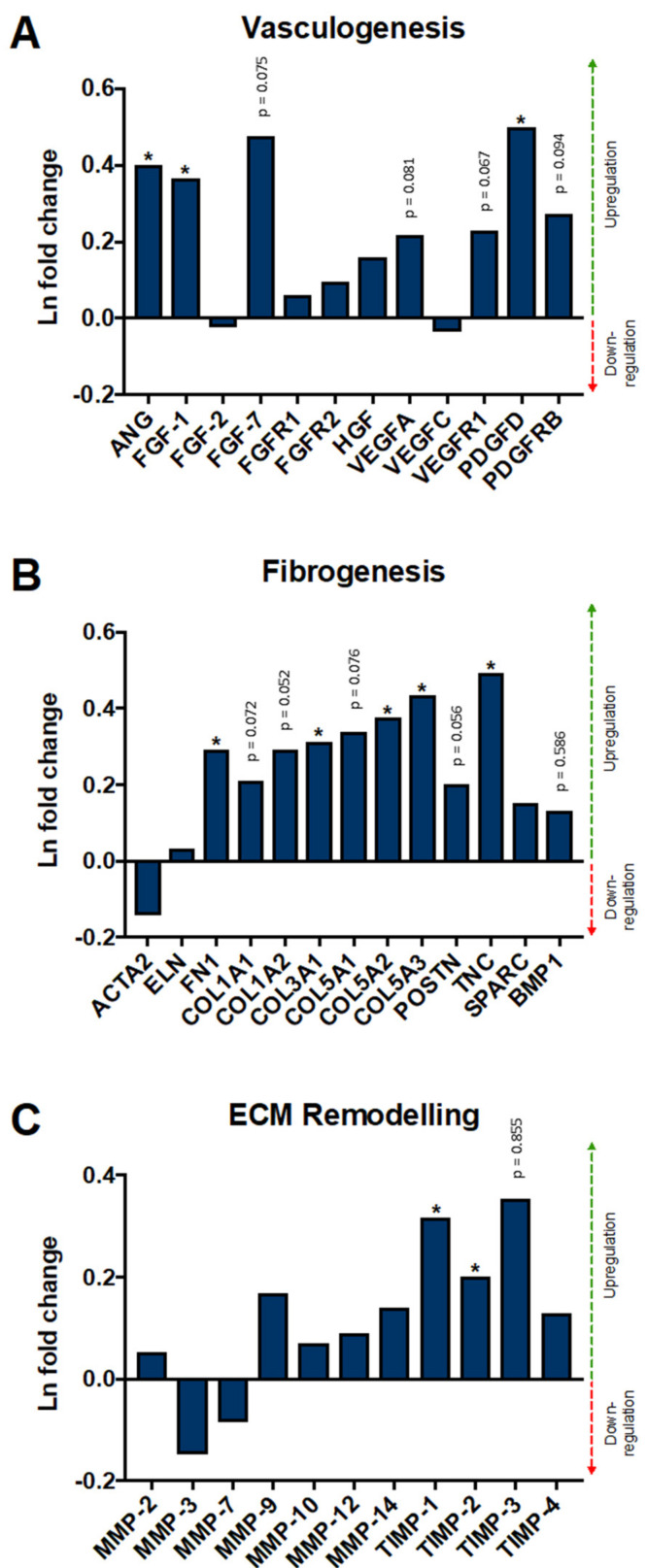
RNA Sequencing of Human Cardiac Fibroblasts on Dye-Mediated Photo-Oxidation (DMPO)-fixed Bioscaffold Relative to Matched Cells on Glutaraldehyde (GA)-Fixed Bioscaffold. (**A**) Selected genes associated with vasculogenesis are upregulated in human cardiac fibroblasts on DMPO-fixed ECM bioscaffold relative to GA-fixed ECM bioscaffold (N = 6/group). (**B**) Selected genes associated with fibrogenesis are upregulated in human cardiac fibroblasts on DMPO-fixed ECM bioscaffold relative to GA-fixed ECM bioscaffold (N = 6/group). (**C**) Selected genes associated with extracellular matrix (ECM) remodeling are differentially expressed in human cardiac fibroblasts on DMPO-fixed ECM bioscaffold relative to GA-fixed ECM bioscaffold (N = 6/group). Values represent mean ± SD. * *p* < 0.05. False discovery rate corrected *p*-values determined using the Benjamin–Hochberg procedure.

Figure 1 shows the differential gene expression of cardiac fibroblasts seeded on DMPO-fixed ECM relative to cardiac fibroblasts seeded on GA-fixed ECM. Genes categorized to key themes of fibroblast activity: vasculogenesis (Figure 1A), fibrogenesis (Figure 1B), and ECM remodeling (Figure 1C). Regarding vasculogenesis, DMPO-fixed ECM induced higher human cardiac fibroblast transcription of angiogenin, fibroblast growth factor-1, and platelet-derived growth factor-D compared to GA-fixed ECM (Figure 1A). Similar phenomena were seen with genes related to fibrogenesis, including fibronectin 1, collagen 3A1, collagen 5A2, collagen 5A3, and tenascin C (Figure 1B). The transcription of genes related to ECM remodeling was similar for both groups regarding examined matrix metalloproteinases (MMPs); however, DMPO-fixed ECM displayed increased transcription of tissue inhibitors of metalloproteinases, including TIMP-1 and TIMP-2 (Figure 1C). Supplemental File S1 shows the complete RNA sequencing data set.

### 2.2. DMPO-Fixed ECM Alters the Secretome of Human Cardiac Fibroblasts to Promote the Release of Vasculogenic Factors

Modulating cellular activity may provide another layer of repair when DMPO-fixed scaffold is implanted. Thus, we wanted to examine this material’s modulatory effect on cardiac fibroblasts and compare it with the effects of SIS-ECM, which is known to be bioactive. Multiplex analysis assessed if DMPO-fixed ECM could alter the secretome of human cardiac fibroblasts similar to a known bioinductive non-fixed porcine-derived small intestinal submucosa ECM, bioactive SIS-ECM. Conditioned media collected after 24 h of culture was used to assess the secretome of human cardiac fibroblasts when seeded on DMPO-fixed ECM, bioactive SIS-ECM, or tissue culture plastic (control). Tissue culture plastic was used as a control to maintain consistency with our previously validated models [10]. This group provided a reference point to compare how DMPO-fixed bovine pericardium alters fibroblast activity to the known bioinductivity of SIS-ECM. 

**Figure 2:**
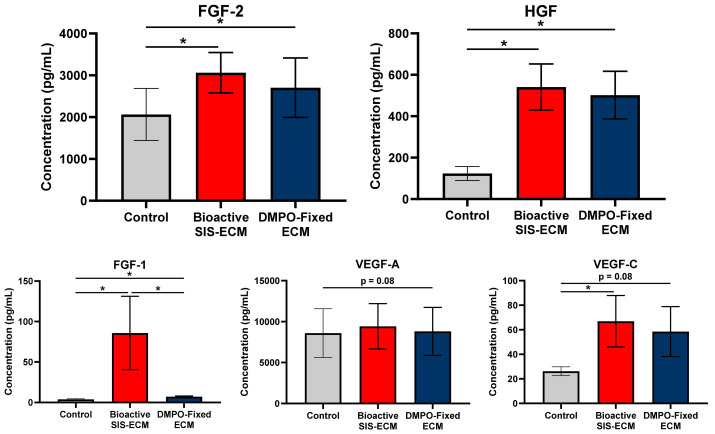
Dye-Mediated Photo-Oxidation (DMPO)-fixed ECM Bioscaffold Promotes a Pro-Vasculogenic Paracrine Response from Human Cardiac Fibroblasts Similar to Bioactive Small Intestinal Submucosa (SIS) ECM Bioscaffold. Multiplex analysis of fibroblast growth factor-2 (FGF-2), hepatocyte growth factor (HGF), fibroblast growth factor-1 (FGF-1), vascular endothelial growth factor-A (VEGF-A), and vascular endothelial growth factor-C (VEGF-C) from conditioned media from human cardiac fibroblasts on tissue culture plastic (control; N = 7), bioactive SIS-ECM (N = 7), or DMPO-fixed ECM bioscaffold (N = 7). Values represent mean ± SEM, baseline-corrected to control. Statistical significance determined by unpaired Student’s *t*-test; symbols denote statistical significance (* = *p*-value < 0.05).

Figure 2 shows the vasculogenic response of cardiac fibroblasts to DMPO-fixed ECM compared to bioactive SIS-ECM. DMPO-fixed ECM increased the relative production of fibroblast growth factor-2, hepatocyte growth factor, and fibroblast growth factor-1, compared to inert tissue culture plastic control (Figure 2). Similar nonsignificant trends were identified for vascular endothelial growth factor-A and vascular endothelial growth factor-C (Figure 2). Compared to bioactive SIS-ECM, other than fibroblast growth factor-1, DMPO-fixed ECM drove a pro-vasculogenic response similar to known bioactive SIS-ECM, including fibroblast growth factor-2, hepatocyte growth factor, vascular endothelial growth factor-A, and vascular endothelial growth factor-C (Figure 2).

**Figure 3:**
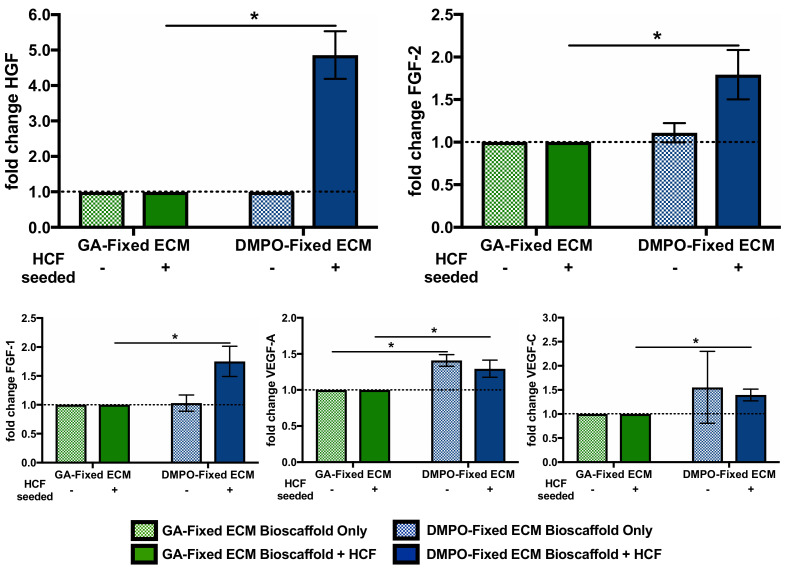
Dye-Mediated Photo-Oxidation (DMPO)-fixed ECM Bioscaffold Promotes a Pro-Vasculogenic Paracrine Response from Human Cardiac Fibroblasts Compared to Glutaraldehyde (GA)-Fixed ECM Bioscaffold. Multiplex analysis of fibroblast growth factor-2 (FGF-2), hepatocyte growth factor (HGF), fibroblast growth factor-1 (FGF-1), vascular endothelial growth factor-A (VEGF-A), and vascular endothelial growth factor-C (VEGF-C) from conditioned media with or without human cardiac fibroblasts (HCF) on GA-Fixed ECM bioscaffold (without HCF, N = 3; with HCF, N = 7) or DMPO-fixed ECM bioscaffold (without HCF, N = 3; with HCF, N = 7). Values represent mean ± SEM. DMPO-fixed ECM bioscaffold only baseline-corrected to GA-Fixed ECM bioscaffold only. DMPO-fixed ECM bioscaffold + HCF baseline corrected to GA-Fixed ECM bioscaffold + HCF. Statistical significance determined by unpaired Student’s *t*-test; symbols denote statistical significance (* = *p*-value < 0.05).

A similar comparison for bioinductivity was performed with GA-fixed bovine pericardium, as it is the most commonly used. Figure 3 shows the aforementioned vasculogenic paracrine factors elicited from DMPO-fixed ECM with and without human cardiac fibroblasts seeded and compared to similar groups, GA-fixed ECM with and without human cardiac fibroblasts seeded. Without cardiac fibroblasts seeded, multiplex analysis was used to quantify vasculogenic paracrine factors eluted from the bioscaffolds into the media; DMPO-fixed ECM eluted more vascular endothelial growth factor-A (Figure 3). With cardiac fibroblasts seeded, a multiplex analysis of DMPO-fixed ECM conditioned media showed increased levels of fibroblast growth factor-2, hepatocyte growth factor, fibroblast growth factor-1, vascular endothelial growth factor-A, and vascular endothelial growth factor-C compared to GA-fixed ECM (Figure 3). When comparing DMPO-fixed ECM groups with and without human cardiac fibroblasts seeded, the increases in fibroblast growth factor-2, hepatocyte growth factor, and fibroblast growth factor-1, were due to bioinductive effects due to the presence and interaction of cardiac fibroblasts. 

### 2.3. DMPO-Fixed ECM Preserves Cardiac Fibroblast Phenotype and Promotes Cellular Viability

Three-dimensional microgel-bioscaffold constructs were used to assess the bioinductive effect of DMPO-fixed ECM, compared to GA-fixed ECM, as it allowed for the characterization of individual cardiac fibroblast cell morphology when exposed to each bioscaffold.

**Figure 4:**
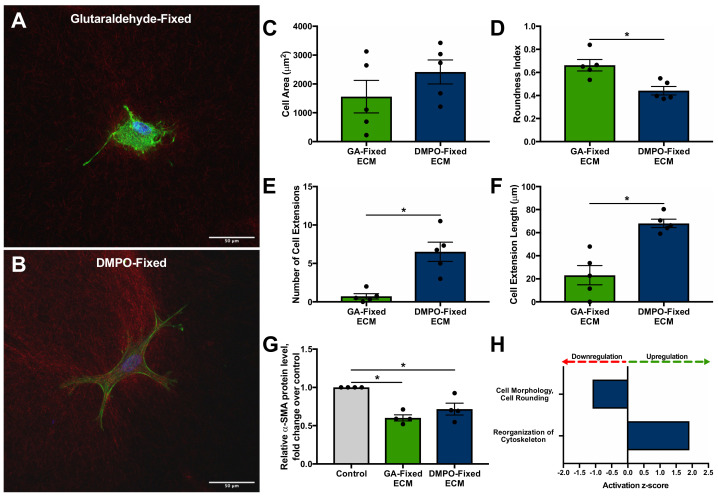
3D Microgel Assessment of Human Cardiac Fibroblasts within Dye-Mediated Photo-Oxidation (DMPO)-fixed ECM Bioscaffold Compared to Matched Cells within Glutaraldehyde (GA)-Fixed ECM Bioscaffold. (**A**,**B**) Representative confocal images of human cardiac fibroblasts within (**A**) GA-fixed ECM bioscaffold and (**B**) DMPO-fixed ECM bioscaffold. (**C**–**F**) Morphological assessment, including cell area, roundness index, number of cell extensions, and cell extension length, of human cardiac fibroblasts within DMPO-fixed ECM bioscaffold or GA-fixed ECM bioscaffold (N = 5 patients/group; 2–5 technical replicates per patient; a total of 16 cells were imaged and analyzed for DMPO-fixed ECM and a total of 18 cells were imaged and analyzed for GA-fixed ECM). Values represent mean ± SEM. Statistical significance determined by unpaired Student’s *t*-test; *p* < 0.05 defined as statistically significant. (**G**) α-smooth muscle actin (αSMA) protein expression measured by Western Blot analysis of human cardiac fibroblasts on tissue culture plastic (Control), GA-fixed ECM bioscaffold, or DMPO-fixed ECM bioscaffold (N = 4/group). Values represent mean ± SEM, baseline-corrected to control. Statistical significance determined by unpaired Student’s *t*-test; *p* < 0.05 defined as statistically significant. (**H**) RNA Sequencing of Human Cardiac Fibroblasts on DMPO-fixed ECM bioscaffold Relative to Matched Cells on Glutaraldehyde (GA)-fixed ECM bioscaffold. Selected pathways and functions are differentially expressed in human cardiac fibroblasts on DMPO-fixed ECM bioscaffold relative to GA-fixed ECM bioscaffold (N = 6/group). Values represent activation z-score (upregulation, activation z-score >0; downregulation, activation z-score < 0). False discovery rate corrected *p*-value < 0.05 for all represented data, determined using the Benjamin–Hochberg procedure for multiple comparisons. * *p* = 0.05.

Figure 4 shows representative confocal microscopy images of human cardiac fibroblasts seeded within GA-fixed ECM (Figure 4A) and DMPO-fixed ECM (Figure 4B). Morphological assessment and quantitative analysis demonstrated the preservation of phenotype of cardiac fibroblasts seeded on DMPO-fixed ECM, including decreased roundness index, increased number and length of cell extensions, and a trend towards increased cell area (Figure 4C–F). Western blot showed similar αSMA production by cardiac fibroblasts seeded on DMPO-fixed ECM and GA-fixed ECM, which for each, was decreased relative to tissue culture plastic (control) (Figure 4G). Selected pathways and functions generated through RNA sequencing analysis revealed differential gene expression regarding increased cytoskeleton reorganization and decreased cell rounding of cardiac fibroblasts seeded on DMPO-fixed ECM compared to GA-fixed ECM (Figure 4H).

**Figure 5:**
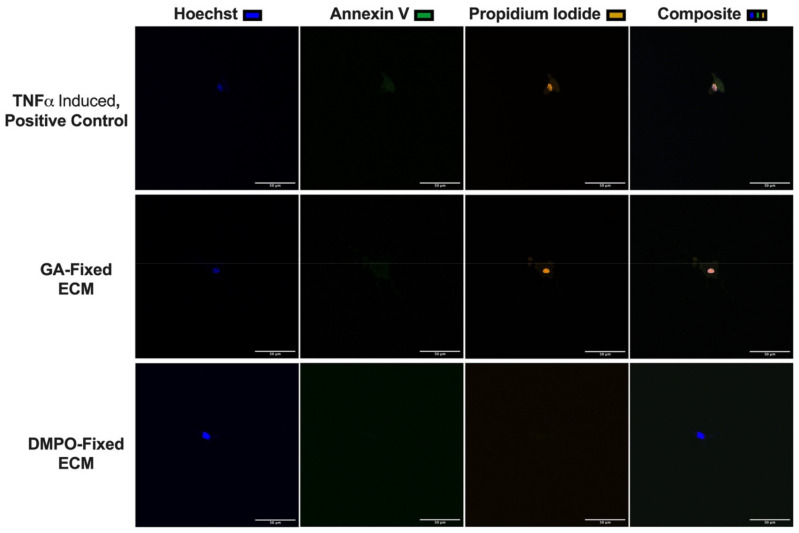
Qualitative 3D Microgel Assessment of Human Cardiac Fibroblasts within Dye-Mediated Photo-Oxidation (DMPO)-fixed ECM Bioscaffold Displays Decreased Cellular Necrosis Compared to Human Cardiac Fibroblasts within Glutaraldehyde (GA)-Fixed ECM Bioscaffold. Representative confocal images of human cardiac fibroblasts within DMPO-fixed ECM bioscaffold, GA-Fixed ECM bioscaffold, or positive control (TNFα-induced, Nylon) (N = 4 patients/group; 5–7 technical replicates per patient; a total of 22 cells were imaged for DMPO-fixed ECM and a total of 20 cells were imaged for GA-fixed ECM, using Hoechst (nuclear stain, blue), annexin V (early apoptosis, green), and propidium iodide (late apoptosis/necrosis, yellow). See Figure A1 for additional representative images.

Confocal microscopy for cellular viability was performed using the same three-dimensional microgel-bioscaffold model. Figure 5 shows representative confocal microscopy images of human cardiac fibroblasts seeded within GA-fixed ECM, DMPO-fixed ECM, or TNFα induced inert nylon-scaffold (positive control). Increased cellular necrosis was demonstrated for cardiac fibroblasts seeded on GA-fixed ECM compared to DMPO-fixed ECM (Figure 5). Representative images from 3 additional patients have been made available in Appendix B.

### 2.4. DMPO-Fixed ECM Demonstrates Greater Compliance Compared to GA-Fixed ECM

**Figure 6:**
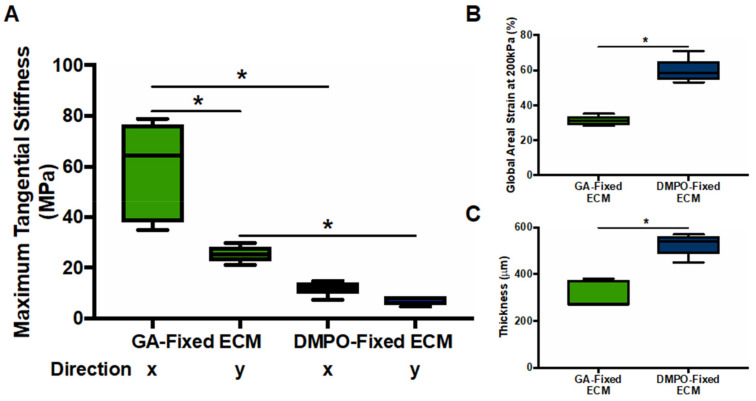
Dye-Mediated Photo-Oxidation (DMPO)-fixed ECM Bioscaffold Displays Increased Compliance Compared to Glutaraldehyde (GA)-Fixed ECM Bioscaffold. (**A**) Maximum tangential stiffness measured by biaxial mechanical testing in the x (horizontal) and y (vertical) directions (GA-fixed ECM, N = 5/group; DMPO-fixed ECM, N = 6/group). Values represent mean ± SEM. Statistical significance determined by one-way ANOVA using the Sidak’s multiple comparisons test; *p* < 0.05 defined as statistically significant. (**B**) Global areal strain measured by biaxial mechanical testing in the x (horizontal) and y (vertical) directions (GA-fixed ECM, N = 5; DMPO-fixed ECM, N = 6). Values represent mean ± SEM. Statistical significance determined by unpaired Student’s *t*-test; *p* < 0.05 defined as statistically significant. (**C**) Bioscaffold thickness measured prior to biaxial mechanical testing (GA-fixed ECM, N = 5; DMPO-fixed ECM, N = 6). Values represent mean ± SEM. Statistical significance determined by unpaired Student’s *t*-test; symbols denote statistical significance (* = *p*-value < 0.05).

Biaxial mechanical testing assessed linear stress-strain relationships (Young’s Modulus or maximal tangential stiffness) to characterize the compliance of DMPO-fixed ECM compared to GA-fixed ECM. Figure 6 shows, in both axes (x-, horizontal; y, vertical), that DMPO-fixed ECM demonstrated lower maximal tangential stiffness (Figure 6A) and higher global areal strain (Figure 6B). DMPO-fixed ECM was also a thicker bioscaffold than GA-fixed ECM (Figure 6C). Biaxial mechanical testing findings demonstrate greater compliance and responsiveness of DMPO-fixed ECM to external stress, with biomechanical properties with a profile more like healthy cardiovascular tissues.

## 3. Discussion

Herein, we compared DMPO-fixed acellular bovine pericardium to the widely used GA-fixed bovine pericardium to evaluate the material’s potential for use in cardiac surgery. Additionally, we compared it to bioactive SIS-ECM as a reference point to assess the material’s bioinductive properties. Our results show that DMPO-fixed acellular bovine pericardium is a fixed material that demonstrates bioinductive properties similar to non-fixed acellular matrix bioscaffolds. The material’s properties are promising for reconstruction and upcoming procedures for post-infarct cardiac repair, such as epicardial implantation over ischemic areas. Dye-mediated photo-oxidation (DMPO)-fixed ECM is a suitable bioscaffold for human cardiac fibroblasts that preserves cellular phenotype, and potentiates a pro-vasculogenic paracrine response that may participate in tissue repair after injury. DMPO-fixed bovine pericardium preserves cell viability seen on confocal imaging, while demonstrating compliance, as shown by biaxial mechanical testing. Given the potential of ECM bioscaffolds in cardiac reconstruction and repair, DMPO-fixed ECM may serve as a viable alternative to glutaraldehyde (GA)-fixed ECM biomaterials because they provide added bioinductivity. Additionally, the material’s structural compliance may suggest that it would not restrict movement of the heart and would not impair diastolic function when applied to the epicardium.

Bioinductivity of DMPO-fixed ECM adds an additional dimension when used in cardiac surgery, as leveraging endogenous cells may enable improved integration. Bioinductive properties of DMPO-fixed ECM suggest that it may potentiate reparative mechanisms when implanted. Cardiac fibroblasts are key regulators of extracellular matrix homeostasis and can contribute to post-infarct neovascularization following acute ischemic injury via mesenchymal-to-endothelial transition and paracrine activity [17,18,19]. Interaction with DMPO-fixed ECM upregulated the pro-vasculogenic paracrine response of cardiac fibroblasts. The roles of vascular endothelial growth factor (VEGF) and fibroblast growth factor (FGF) in promoting angiogenesis and enhancing myocardial perfusion have been well characterized [10,20,21,22,23]. Hepatocyte growth factor (HGF) has not only been shown to directly induced angiogenesis following myocardial infarction, but also plays a synergistic role in enhancing the effects of VEGF mediated angiogenesis [24,25,26,27].

The expression of tissue inhibitors of metalloproteinases, including TIMP-1 and TIMP-2, was also modulated by DMPO-fixed ECM. Tissue inhibitors of metalloproteinases are involved in direct and indirect ECM regulation, and the altering of the ratio of TIMPs to their matrix metalloproteinase counterparts shifts ECM homeostasis [16,28]. Investigations of TIMP-1 have shown attenuated maladaptive cardiac remodeling at the site of myocardial infarction, inhibition of cardiomyocyte apoptosis, and enhanced pro-vasculogenic factor release from cardiac fibroblasts, including VEGF, FGF, and HGF [29]. Our group has previously characterized the concentration-dependent interaction amongst TIMP-2 and human cardiac fibroblasts; the role of TIMP-2 in stimulating ECM turnover while attenuating maladaptive cardiac remodeling has also been reported [30,31]. Additionally, DMPO-fixed ECM altered the fibroblast transcription of fibrogenic ECM components, including fibronectin 1 (FN1), collagen 3A1 (COL3A1), collagen 5A2 (COL5A2), collagen 5A3 (COL5A3), and tenascin C (TNC). Given the modulation of vasculogenic, fibrogenic, and ECM remodeling genes, in addition to the increased production of pro-vasculogenic paracrine factors by cardiac fibroblasts, this suggests that DMPO-fixed ECM bioscaffold better preserves and regulates cardiac fibroblast homeostatic function. Moreover, DMPO-fixed ECM may provide an opportunity for enhanced bioscaffold directed cardiac repair upon implantation, given its ability to modulate ECM remodeling and potentiate a pro-vasculogenic response.

Given its reconstructive nature, cardiac surgery heavily relies on GA-fixed ECM bioscaffolds for robust and reproducible repair in a variety of cases including valve repair, repair of septal defects, vascular reconstruction, pediatric cardiac surgery, and other indications. The use of GA-fixed ECM bioscaffolds remains suboptimal, given the associated complications, including implant calcification, inflammatory reaction, glutaraldehyde cytotoxicity, and overall poor bioscaffold-host tissue integration [1,3,6,32,33,34,35,36]. In comparison, DMPO-fixed ECM preserves cardiac fibroblast phenotype, as indicated by morphological assessment. DMPO-fixed ECM thereby demonstrates enhanced biocompatibility compared to GA-fixed ECM bioscaffold. Furthermore, the stability of GA-fixed ECM is a key advantage to its use, while calcification is a key disadvantage. Increased stability has been shown to be a result of greater glutaraldehyde uptake and concomitant increased collagen cross-linking [1]. However, increased glutaraldehyde uptake has also been shown to exacerbate bioscaffold calcification [1]. These challenges associated with GA-fixed ECM bioscaffolds have been shown to result in poor bioscaffold-host cell integration and restricted angiogenesis at the site of implantation [6]. DMPO-fixed ECM bioscaffold has been shown to be non-calcific and non-immunogenic when compared to GA-fixed ECM bioscaffold [37,38,39]. Use of DMPO-fixed ECM in pediatric cardiac surgery revealed absent or minimal inflammation and calcification at the site of implantation; partial endothelialization of DMPO-fixed ECM has also been previously described [38,40]. Given that DMPO-fixed ECM supports not only healthy cardiac fibroblast homeostatic function, but also promotes a pro-vasculogenic response, this may contribute to enhanced bioscaffold-host tissue integration. The role of controlled VEGF expression has previously been shown to improve engineered cardiac bioscaffold integration, due to increased cellular viability and bioscaffold-host tissue neovascularization [41].

Regarding mechanical properties, GA-fixed ECM used for vascular reconstruction has previously been shown to display increased stiffness compared to native tissue [42]. While increased stiffness may provide robust structural repair, limited compliance has also been shown to contribute to graft failure [43]. Poor compliance has also been proposed to contribute to diastolic dysfunction in the context of bioscaffolds for ventricular restraint [44]. DMPO-fixed ECM may circumvent these challenges given its enhanced compliance, while still providing a robust and predictable bioscaffold option given its fixation. Therefore, in addition to its non-immunogenic and non-calcific properties previously described, the enhanced biocompatibility, mechanical properties, and pro-vasculogenic response that we now highlight, may together support the bioscaffold-host tissue integration of DMPO-fixed ECM.

A potential novel use for DMPO-fixed ECM bioscaffold may be for post-infarct cardiac repair via epicardial implantation, whereby biomaterial patches are sewn over ischemic myocardial tissue during coronary artery bypass surgery to promote reparative activity. Our group has previously leveraged ECM bioscaffolds to enhance cardiac repair following myocardial infarction. Our prior work demonstrated enhanced vasculogenesis and functional myocardial recovery at the site of myocardial infarction using non-fixed bioactive SIS-ECM bioscaffold [9,10,11]. An enhanced FGF-2-dependent cell-mediated paracrine response was shown to be critical to the bioinductive effects of SIS-ECM [9,10,45]. While advantageous in their ability to drive endogenous cellular activity, immunogenicity and the elicitation of an inflammatory response upon surgical implantation present challenges to the clinical translation of non-fixed SIS-ECM bioscaffolds [12,13]. DMPO-fixed ECM elicited a similar bioinductive pro-vasculogenic paracrine response to that of bioactive SIS-ECM, including FGF-2, VEGF-A, VEGF-C, and HGF. Additionally, the non-immunogenic character of DMPO-fixed ECM has previously been described [37,38,39]. The potential of DMPO-fixed ECM therefore again extends past enhanced biocompatibility, as it also displays bioinductive properties capable of modulating the paracrine response of cardiac fibroblasts, similar to that of a known bioactive ECM bioscaffold. 

Biocompatibility, differential mechanical properties, and bioinductive effects of DMPO-fixed ECM make it a viable alternative to glutaraldehyde (GA)-fixed ECM bioscaffold. This material provides structural support and demonstrates the added benefit of bioactivity. Together, findings may support DMPO-fixed ECM bioscaffold-host tissue integration and utility in epicardial infarct repair, among other reparative and reconstructive indications within the scope of cardiac surgery.

We acknowledge the limitations of this study, given that endogenous bioscaffold-host tissue integration is a complex multifaceted process driven by many processes, we assess only a limited subset of these processes, including ECM remodelling and vasculogenic response. As such, in vivo assessment of functional ECM remodelling and neovascularization is a necessary next step in characterizing the effects of DMPO-fixed ECM bioscaffold. Secondly, once in vivo correlates are developed, it may be beneficial to mechanistically characterize cell-bioscaffold interactions to identify key pathways that can be leveraged to promote biocompatibility and bioinductive effects of DMPO-fixed ECM, and future iterations of biomaterials used in cardiac surgery. Future directions could potentially include data on degradation, non-thrombogenicity and eluted factors to further evaluate the material for safety.

## 4. Materials and Methods

### 4.1. Biomaterials

All biomaterials used in the above studies are commercially available. Dye-mediated photo-oxidation fixed bovine pericardium (PhotoFix; CryoLife Inc., Kennesaw, GA, USA) was compared with unfixed lyophilized acellular porcine small intestina submucosal extracellular matrix (SIS-ECM; Cor^TM^ PATCH; CorMatrix Cardiovascular Inc., Roswell, GA, USA) and glutaraldehyde-fixed bovine pericardium (Peri-Guard; Synovis, MN, USA). All materials are commercially available; therefore, preparation methods are proprietary.

### 4.2. Isolation and Expansion of Human Cardiac Fibroblasts

Human cardiac fibroblasts were isolated from the right atrial appendage of consenting patients undergoing cardiac surgery at the Foothills Medical Center (Calgary, AB, Canada), as previously described [9,10]. Tissue was processed into 0.5 mm–1.0 mm pieces and cultured in Iscove’s modified Dulbecco’s medium (IMDM; Lonza, MD, USA) supplemented with 10% fetal bovine serum (Gibco by Life Technologies, Burlington, ON, Canada) and 50,000 units of penicillin-streptomycin (Life Technologies, Burlington, ON, Canada). Cells were cultured in 75 cm^2^ flasks coated with 0.1% gelatin at 37 °C in 5% CO_2_. Cells were serum-starved for 24 h prior to use; and E0P1–E0P5 were used for experiments. See Appendix A for patient characteristics. Dye-mediated photo-oxidation fixed bovine pericardium (DMPO-fixed ECM), PhotoFix^®^ (Cryolife, Inc., Kennesaw, GA, USA). Glutaraldehyde-fixed bovine pericardium (GA-fixed ECM), Peri-Guard^®^ (Synovis Life Technologies, St. Paul, MN, USA). Non-fixed small intestinal submucosa (Bioactive SIS-ECM), CorMatrix^®^ (CorMatrix Cardiovascular Inc., Roswell, GA, USA). Ethical approval for this study was obtained from the Conjoint Health Research Ethics Board (CHREB) at the University of Calgary.

### 4.3. RNA Sequencing Analysis of Human Cardiac Fibroblasts on Bioscaffolds

Circular punchouts 12 mm in diameter of each bioscaffold, DMPO-fixed ECM and GA-fixed ECM, were presoaked in PBS for 30 min. Using 24-well plates, one hundred thousand human cardiac fibroblasts were seeded onto each bioscaffold, and then incubated in a total of 500 µL of serum-free IMDM at 37 °C in 5% CO_2_ for 24 h. Total RNA was isolated from human cardiac fibroblasts seeded on each bioscaffold. RNA integrity was evaluated using Agilent 2200 Tapestation RNA (RIN) assay (Agilent, CA, USA). From the isolate, 30 µL was used for cDNA library preparation [TruSeq Stranded mRNA Library Preparation (Illumina, CA, USA)]. RNA sequencing data were generated (535 M reads) using a 75-cycle high-output kit on an Illumina NextSeq500 (Illumina, CA, USA). RNA sequencing reads were pseudo-aligned to the human NCBI RefSeq transcript database dated January 2017, using Kallisto 0.42.4 [46,47]. For parametric analysis, Sleuth was used for differential gene expression using a linear model containing two terms: the nominal scaffold factor and the patient from which each sample was derived [48]. Genes or transcripts passing the likelihood ratio test with Benjamin–Hochberg procedure (false discovery rate) corrected *p*-values < 0.05 were considered differentially expressed. Differentially expressed genes were annotated and analyzed for enrichment using ingenuity pathway analysis (Qiagen, Germantown, MD, USA).

### 4.4. Biochemical Characterization of Bioscaffolds

Elution studies identified relevant angiogenic factors released from each bioscaffold, if any. Circular punchouts 12 mm in diameter of each bioscaffold, DMPO-fixed ECM and GA-fixed ECM, were incubated in serum-free IMDM media for 24 h at 37 °C in 5% CO_2_ for bioactive factors to elute into the media. The conditioned elution media was analyzed via multiplex analysis (Eve Technologies, Calgary, AB, Canada) to quantify fibroblast growth factor-2 (FGF-2), fibroblast growth factor-1 (FGF-1), hepatocyte growth factor (HGF), vascular endothelial growth factor-A (VEGF-A), and vascular endothelial growth factor-C (VEGF-C) released from the bioscaffolds.

### 4.5. Characterization of the Human Cardiac Fibroblast Paracrine Response to Bioscaffolds

Circular punchouts 12 mm in diameter of each bioscaffold, DMPO-fixed ECM, GA-fixed ECM, and bioactive SIS-ECM, were presoaked in PBS for 30 min. Using 24-well plates, one hundred thousand human cardiac fibroblasts were seeded onto each punchout, or cell culture plastic (control), then incubated in a total of 500 µL of serum-free IMDM at 37 °C in 5% CO_2_ for 24 h. Conditioned media were then collected and analyzed via multiplex (Eve Technologies, Calgary, AB, Canada) to quantify FGF-2, FGF-1, HGF, VEGF-A, and VEGF-C from human cardiac fibroblasts interacting with these bioscaffolds.

### 4.6. Characterization of Human Cardiac Fibroblast Cell Morphology and Viability Using a Bioscaffold-Microgel Model

Our group has previously described the preparation and imaging of a novel 3D collagen microgel-bioscaffold model for the assessment of human cardiac fibroblast morphology [9]. Human cardiac fibroblasts were seeded onto bioscaffold-collagen microgel constructs, made using either DMPO-fixed ECM or GA-fixed ECM, and a morphological and viability assessment was carried out. Using 24-well plates, a low density of cells (5000 cells/well) was used to allow for single-cell characterization in this model. For human cardiac fibroblast morphology assessment, cells were seeded for 24 h, constructs were fixed in 4% PFA and permeabilized using 0.1% Triton-X. F-actin cytoskeleton was visualized with Alexa Fluor 488 phalloidin staining (Life Technologies, Burlington, ON, Canada) and nuclei were stained with Hoechst. Morphologic assessment included cell area, number of cell extensions, cell extension length, and cell roundness, as previously described by our group and others [9,49,50]. All cells were randomly selected. Cell extension length was assessed from the center of the cell to the tip of the extension, using ImageJ image analysis software (Ver. 1.50, NIH, USA). Cell extensions were defined as any primary cell extension greater than 15 µm in length originating from the cell body or any secondary cell extension greater than 15 um in length originating from a primary extension. Multi-Cell Outliner ImageJ plug-in (http://rsbweb.nih.gov/ij/plugins/multi-cell-outliner.html; accessed on 1 February 2019) was used to quantify cell roundness (value = 1.0, indicates a perfect circle) and cell area. For human cardiac fibroblast qualitative viability assessment, cells were seeded for 24 h, and visualized using an Annexin-V Propidium Iodide apoptosis kit (Thermo Fisher Scientific, Waltham, MA, USA); nuclei were stained with Hoechst. TNFα (250 ng/mL) was used to induced apoptosis in the positive control group. Constructs were imaged using a confocal microscope (LSM 5, Carl Zeiss, Oberkochen, Germany).

### 4.7. Western Blot Analysis of Alpha-Smooth Muscle Actin of Human Cardiac Fibroblast on Bioscaffolds

Circular punchouts 12 mm in diameter of each bioscaffold, DMPO-fixed ECM and GA-fixed ECM, were presoaked in PBS for 30 min. Using 24-well plates, one hundred thousand human cardiac fibroblasts were seeded onto each bioscaffold, or cell culture plastic (control), then incubated in a total of 500 µL of serum-free IMDM at 37 °C in 5% CO_2_ for 24 h. Protein was isolated from human cardiac fibroblasts and combined with 4X NuPAGE LDS sample buffer (Invitrogen, Waltham, MA, USA). Moreover, 20 uL of each sample was run on 15% SDS-PAGE gel at 200 V for 135 min, and then transferred onto a nitrocellulose membrane at 100 V for 90 min (Millipore, Temecula, LA, USA). The nitrocellulose membrane was blocked with 5% skim milk in tris-buffered saline +0.05% tween (TBST) for 1 h at room temperature. The membrane was incubated with mouse anti-αSMA antibody diluted 5:10,000 in 5% skim milk in TBST (Sigma Aldrich, St. Louis, MO, USA) overnight at 4 °C. After washing, the membrane was incubated with goat anti-mouse IgG horseradish peroxidase-linked antibody diluted 5:10,000 in 5% skim milk in TBST for 1 h at room temperature (Thermo Fisher Scientific, Waltham, MA, USA). After washing, the membrane was incubated for 1 min in luminol and peroxide (SuperSignal West Femto Maximum Sensitivity Substrate; Thermo Fisher Scientific, Waltham, MA, USA). A chemiluminescence imaging machine was used for signal detection. The nitrocellulose membrane was then incubated with mouse anti-GAPDH antibody diluted 5:10,000 in 5% skim milk in TBST (primary antibody; Santa Cruz Biotechnology, Dallas, TX, USA) and goat anti-mouse IgG horseradish peroxidase-linked antibody diluted 5:10,000 in 5% skim milk in TBST (secondary antibody; Thermo Fisher Scientific, Waltham, MA, USA), as stated above.

### 4.8. Biomechanical Characterization of Bioscaffolds

Biomechanical characterization was performed by assessing stress-strain relationships (Maximum Tangential Stiffness/Young’s Modulus) for each bioscaffold [9]. Furthermore, 12 mm × 12 mm samples of each bioscaffold, DMPO-fixed ECM and GA-fixed ECM, were immersed in phosphate buffer solution (PBS) for 10 min before testing. The samples were mounted on a biaxial testing machine (ElectroForce Systems, TA Instruments, New Cascle, DE, USA) by attaching suture lines to each, using four fish hooks per side, allowing the machine to stretch each side in two orthogonal planes of stress (x-, horizontal; y-, vertical). Dots were drawn on each material in a predetermined pattern and tracked by digital video extensometer camera for local displacement and global areal strain measurement. Sample thickness was recorded prior to initiating the loading protocol. Regarding the loading protocol, samples were immersed in PBS at 37 °C and pre-loaded to the range of 0.05–0.07 N, then tested using displacement-controlled loading to 40% strain for 10 loading/unloading cycles (preconditioning for 9 cycles followed by generation of strain-stress curve with final cycle) [51]. Test protocols were carried out at peak strain ratios between the two orthogonal planes of stress of 1:1, 1:0.75, 1:0.5, 0.5:1, and 0.75:1 for each sample. The 1:1 protocol was then repeated to detect damage to the sample.

### 4.9. Statistical Analysis

All data are expressed as mean ± SEM of independent samples. Data assumed to be normally distributed were analyzed with unpaired *t*-tests. Other data were analyzed with Wilcoxon sign rank tests. Prism 7.0d (GraphPad Software Inc., San Diego, CA, USA) was used for all statistical analysis. Student *t*-test was used for analysis with an alpha of 0.05. For RNA sequencing analysis, likelihood ratio test with Benjamin–Hochberg procedure (false discovery rate) corrected *p*-values < 0.05 were considered for differential expression.

## 5. Conclusions

Dye-mediated photo-oxidation (DMPO)-fixed ECM is a fixed bioscaffold that demonstrates bioinductive properties and favorable structural characteristics, such as more physiologic tissue compliance on biaxial testing. This biomaterial supports healthy human cardiac fibroblast activity and provides a biocompatible environment compared to glutaraldehyde (GA)-fixed ECM. Given its previously described non-immunogenic and non-calcific properties, and our findings related to bioinductive and mechanical properties, DMPO-fixed ECM should be further investigated as a viable tool for cardiac reconstruction and post-infarct repair.

## Figures and Tables

**Figure A1:**
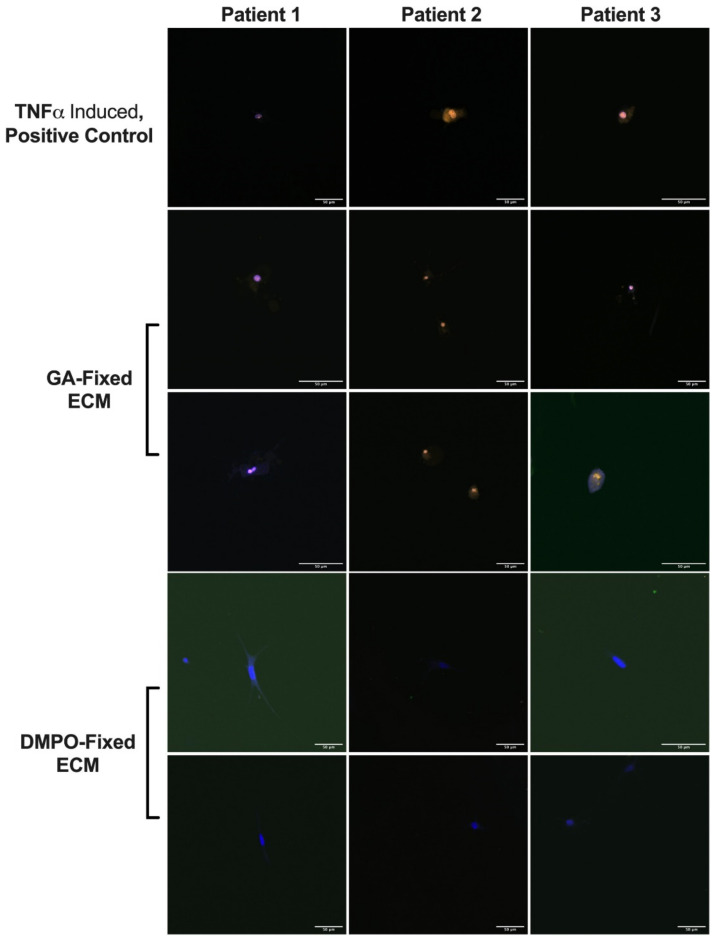
Additional Representative Images for Qualitative 3D Microgel Assessment of Human Cardiac Fibroblasts within Dye-Mediated Photo-Oxidation (DMPO)-fixed ECM Bioscaffold Displaying Decreased Cellular Necrosis Compared to Human Cardiac Fibroblasts within Glutaraldehyde (GA)-Fixed ECM Bioscaffold. Representative confocal images of human cardiac fibroblasts within DMPO-fixed ECM bioscaffold, GA-Fixed ECM bioscaffold, or positive control (TNFα-induced, Nylon) (N = 4 patients/group; 5–7 technical replicates per patient; a total of 22 cells were imaged for DMPO-fixed ECM and a total of 20 cells were imaged for GA-fixed ECM), using Hoechst (nuclear stain, blue), annexin V (early apoptosis, green), and propidium iodide (late apoptosis/necrosis, yellow).

## Data Availability

Data is available upon request to corresponding author.

## Supplementary Materials

The following are available online at https://www.mdpi.com/article/10.3390/ijms221910768/s1.

## Appendix A

**Table A1:** Patient demographic data for right atrial appendage tissue collection from consenting patients undergoing cardiac surgery at the Foothills Medical Center (Calgary, AB, Canada).

Patient Demographics		N = 13
Sex	M	10 (76.9%)
	F	3 (23.1%)
Age (years)	Average Age	64.3
	>65	6 (46.2%)
Surgical Procedure	CABG	6 (46.2%)
	CABG + AVR	2 (15.4%)
	CABG + MVR	0 (0.0%)
	AVR	5 (38.5%)
	MVR	0 (0.0%)
Cardiovascular Risk Factors	HTN	8 (61.5%)
	Dyslipidemia	8 (61.5%)
	DM	4 (30.8%)
	Smoking History	4 (30.8%)
Co-existing Conditions	CAD	8 (61.5%)
	ACS	4 (30.8%)
	Renal Insufficiency	2 (15.4%)

CABG, coronary artery bypass graft; AVR, aortic valve replacement; MVR, mitral valve replacement; HTN, hypertension; DM, diabetes mellitus; CAD, coronary artery disease; ACS, acute coronary syndrome.
